# The impact of the timing of spinal decompression on urinary and sexual function after acute spinal cord injury

**DOI:** 10.1002/bco2.70163

**Published:** 2026-01-30

**Authors:** Matthew Playfair, J. Andrew McClure, Chris Bailey, Blayne Welk

**Affiliations:** ^1^ Western University London ON Canada; ^2^ London Health Sciences Center London ON Canada

**Keywords:** neurogenic, quality of life, sexual health, spinal cord injury, urinary bladder, urology

## Abstract

**Introduction:**

While earlier decompression after spinal cord injury (SCI) is linked to better motor recovery, its impact on bladder and sexual function remains unexplored. Our objective was to determine if time to surgical decompression is associated with bladder and sexual function.

**Methods:**

We conducted a retrospective cohort study using the prospectively collected Canadian Rick Hansen SCI Registry. Primary exposure was time to surgical decompression. Primary outcome was abnormal bladder function defined by use of catheters or any incontinence at 1‐year. Secondary outcomes were sexual function and motor score. Adjusted logarithmic regression models were used.

**Results:**

One thousand thirty‐eight participants met inclusion criteria. Median time to surgical decompression was 25 (IQR17–50) hours, and 46% (475/1038) had early decompression (<24 h). There were 63% (650/1038) who had evidence of abnormal bladder function at 1‐year. On multivariate regression, time to decompression was not significantly related to abnormal bladder function (OR 1.00, 95% CI 1.00–1.01, *p* = 0.38); older age (OR 1.13, 95% CI 1.03–1.23, *p* = 0.01) and worse ASIA score (ASIA A OR 16.35, *p* < 0.01, ASIA B OR 5.12, *p* < 0.01 and ASIA C OR 2.23, *p* < 0.01 all relative to ASIA D) were significantly associated with abnormal bladder function. These results were similar in several sensitivity analyses. Time to decompression was also not significantly associated with sexual function or motor score at 1‐year.

**Conclusions:**

A shorter time to surgical decompression after SCI was not associated with improved bladder or sexual function outcomes; however, older age and a more complete injury were significant predictors.

## 1 INTRODUCTION

In North America, it is estimated that there are 39 new acute spinal cord injuries (SCI) per million people annually.^1^ The initial management of traumatic SCI consists of medical optimisation and then in most cases spinal decompression and stabilization in the operating room. After rehabilitation, there are several important secondary health complications of SCI, such as spasticity, pain, pressure sores and genitourinary dysfunction. While no longer the primary cause of death in people with SCI, neurogenic lower urinary tract dysfunction (NLUTD) and its complications continue to have a significant impact.^2^, ^3^, ^4^ For example, urinary complications are the second most common reason for hospitalization, and bladder management has been identified as the leading health concern by people with SCI.^4^, ^5^ Sexual dysfunction after SCI includes challenges with erectile function, ejaculation and sexual satisfaction.^6^

Appropriate bladder and sexual function rely on a complex interplay of sacral parasympathetic fibres, thoracolumbar sympathetic fibres and somatic innervation, which are all susceptible to damage after SCI.^3^, ^7^ The level and completeness of a SCI can have a significant influence on NLUTD/sexual function and the possible treatment options. The true extent of NLUTD is often unknown until the resolution of the spinal shock phase and may change even years after SCI.^7^, ^8^ Two important factors that impact the potential for recovery and long‐term severity of a SCI are the initial degree of the neurologic damage and the time to spinal decompression. Time to decompression has been studied primarily in motor recovery outcomes; however, its effect on NLUTD and sexual function outcomes has not been clarified.^9^ Intuitively, the timing of spinal cord decompression would also influence NLUTD severity.

The objective of this study is to use a national, multi‐institutional SCI registry database to determine if the time to spinal cord decompression/stabilization is associated with 1‐year post‐SCI bladder, sexual and motor function.

## 2 METHODS

This is a retrospective cohort study that was performed using prospectively collected data. Our hypothesis was that a shorter time to spinal cord decompression/stabilization would be associated with a lower likelihood of bladder, sexual and motor dysfunction.

### 2.1 Data source

The Canada‐wide Rick Hansen Spinal Cord Injury Registry (RHSCIR) was launched in 2004.^10^ It captures data from SCI patients at 30 major trauma and rehabilitation centres across Canada and includes data from the pre‐hospital, acute, rehabilitation and community phases of care. The community follow‐up visits are scheduled at 1, 2 and 5 years and then every additional 5 years. This high‐quality, prospective longitudinal database has been used in multiple research studies in SCI.^11^ All participants included in this study provided written consent, and ethics approval is maintained by all participating sites.

### 2.2 Patient cohort

We included all SCI participants registered in the RHSCIR between 2004 and 2020 who underwent spinal decompression/stabilization surgery for a cervical or thoracic spinal cord injury or cauda equina injury. We excluded people who were less than 18 years of age, those who we could not calculate time between SCI and decompression/stabilization, and those who did not have 1‐year follow‐up data available.

### 2.3 Primary and secondary outcomes

The primary outcome was evidence of abnormal bladder dysfunction at 1 year after SCI. This was defined as use of a urinary catheter (indwelling or intermittent), or an external drainage device; abnormal bladder function on the Functional Independence Measure (FIM) or Spinal Cord Injury Measure (SCIM) question, or self‐reported presence of urinary incontinence.^12^ The FIM and SCIM are validated global assessments of functional domains relevant to people with SCI.^13^ We had two secondary outcomes: evidence of abnormal sexual dysfunction, which was measured with a single question asking about the presence or absence of sexual dysfunction, and motor function, which was measured by the FIM motor score. Further details on these outcomes are included in the online eMethods.

### 2.4 Primary exposure

Our primary exposure was the time between the injury (based on the actual estimate of trauma time) and the half‐way point of the spinal decompression procedure. As per convention, we considered <24 h as early, and ≥24 h as late.^9^

### 2.5 Other variables

Comorbidity count was collected from patient charts at the time of initial assessment. Level of injury and energy level of injury were based on the initial trauma assessment. Trauma severity was assessed using the validated Injury Severity Score (ISS).^14^ American Society of Anesthesiology (ASA) class represents the general morbidity of the patient.^15^ The American Spinal Injury Association (ASIA) classification was used to assess level of motor injury at initial assessment.^16^ In addition, we also calculated the lower extremity motor score, which is an assessment of the motor function (from 0 to 5) of the hip flexors, knee extensors, ankle dorsi and plantar flexors and big toe extensors.

### 2.6 Statistical methods

We compared baseline variables between participants who underwent spinal decompression within 24 h and greater than 24 h using chi‐square tests for categorical variables and Kruskal–Wallis tests for medians. Logistic and linear regression models were used to investigate the impact of time from SCI to decompression on bladder function, sexual function and FIM motor score. Time to decompression was modelled as a continuous variable. The following covariates were included in each model: age, neurological level of injury, energy level that was associated with the injury, ASA, ASIA grade and ISS score. Because of the small number of participants in some groups, sacral level injuries (*n* = 7) were combined with lumbar level injuries and ASA 5 (*n* = 5) was combined with ASA 4. Handling of missing data is described in the eMethods.

To evaluate the strength of the primary study outcome, we conducted several sensitivity analyses, which are fully described in the eMethods. For all analyses, reported *p*‐values are from 2‐tailed tests and a value of <0.05 was considered statistically significant. All statistical analyses were performed using SAS EG version 7.15 (SAS Institute, Cary, North Carolina).

## 3 RESULTS

**FIGURE 1:**
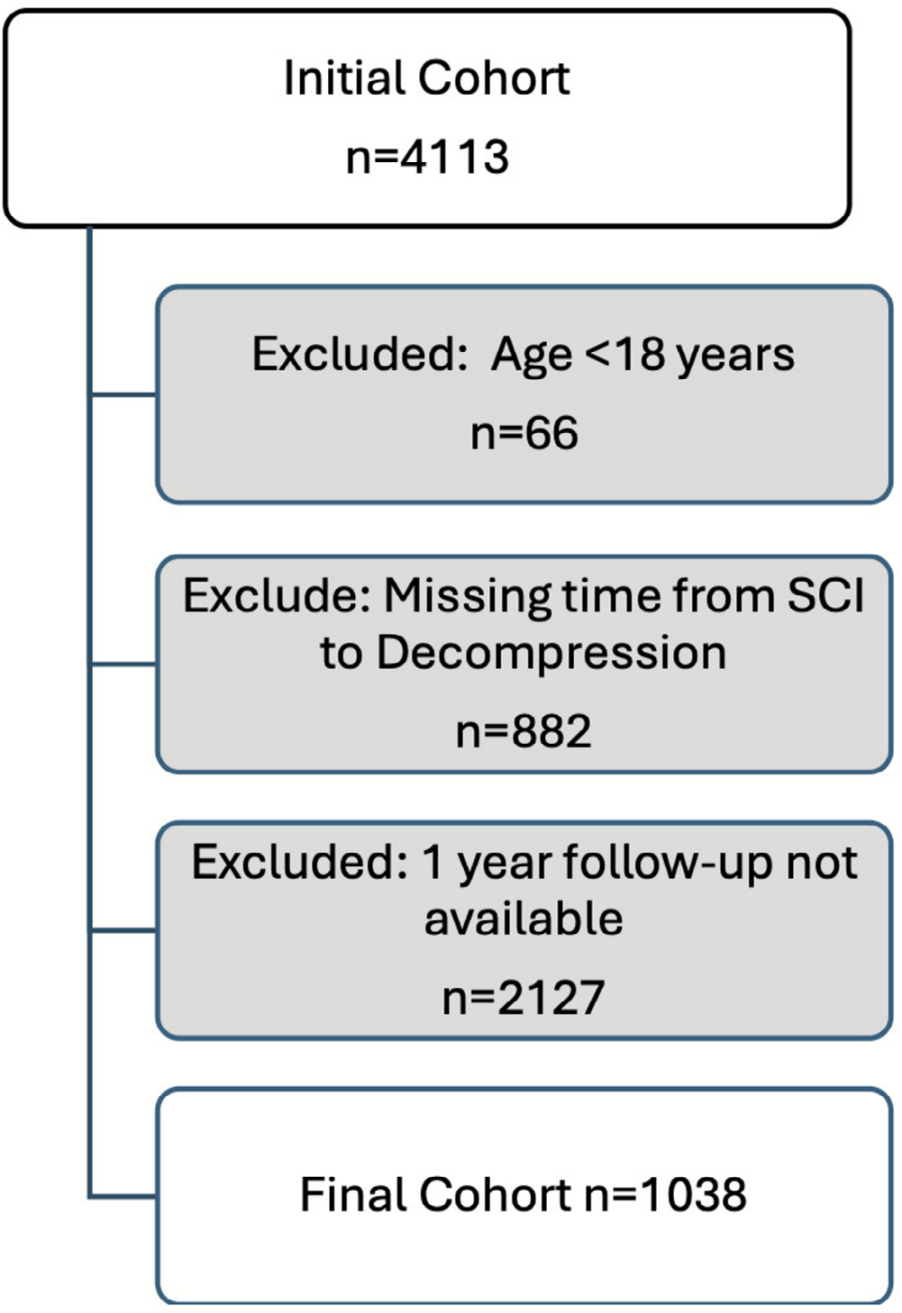
Study design and recruitment. SCI indicates spinal cord injury.

There were 4113 people in RHISCIR who underwent spinal decompression/stabilization and after exclusions 1038 participants remained (Figure 1). At baseline, our cohort had a median age of 46 years (IQR 29–60), 76% were male, and they were generally healthy with a median of 1 (IQR 0–1) comorbidity (Table 1). The most common mechanism of injury was a fall (*n* = 413/1038, 40%), followed by motor vehicle related (*n* = 345/1038, 33%). There were 113 people (11%) who required either air or a combination of air and ground transport to a trauma centre. Additional injuries were common, with a median Injury Severity Score of 24 (IQR 17–30). The SCI level was primarily cervical (*n* = 495/1038, 48%) or thoracic (*n* = 265/1038, 26%). Of those with an ASIA score, about 1/3 had a complete injury (ASIA A) at presentation (*n* = 352/941, 37%). Those who underwent early spinal decompression were younger and had fewer comorbidities. Their injuries were more likely to be high energy with a higher ISS score and ASA class, and they had a higher proportion with complete SCI.

**TABLE 1:** Baseline demographics.

Variable	Value	Early decompression (<24 h)*n* = 475		Late decompression (>24 h)*n* = 563		*p*‐value
Demographics						
Age at SCI	Median (IQR), years	38	(27–56)	50	(34–63)	<0.0001
Sex	Male	353	74.32%	441	78.33	0.129
	Female	122	25.68%	122	21.67%	
Marital Status	Common Law/married	241	50.74%	299	53.11%	0.346
	Divorced/separated/widowed	40	8.42%	55	9.77%	
	Single	164	34.53%	178	31.62%	
	Missing or unknown	30	6.32%	31	5.51%	
Living setting	Private residence	439	92.42%	523	92.90%	0.596
	Assisted living	NR	NR	NR	NR	
	Other	NR	NR	NR	NR	
	Missing or unknown	30	6.32%	30	5.33%	
Facility	British Colombia	163	34.32%	156	27.71%	0.003
	Ontario	92	19.37%	85	15.1%	
	Quebec	64	13.47%	83	14.74%	
	Alberta	34	7.16%	75	13.32%	
	Saskatchewan	15	3.16%	23	4.09%	
	Nova Scotia	18	3.79%	14	2.49%	
	Manitoba	NR	NR	NR	NR	
	New Brunswick	NR	NR	NR	NR	
	Missing	82	17.26%	117	20.78%	
Comorbidity count	Median (IQR)	0	(0–1)	1	(1–1)	0.11
Injury details						
Time from SCI to decompression	Median (IQR), hours	16.5	(11.5–20.5)	47.5	(30.5–93.5)	<0.001
Mechanism of injury	Fall	168	35.37%	245	43.52%	0.076
	Transport	166	34.95%	179	31.79%	
	Sports	103	21.68%	94	16.70%	
	Assault	13	2.74%	15	2.66%	
	Other trauma	25	5.26%	30	5.33%	
Energy level of injury	High	275	57.89%	277	49.20%	0.015
	Low	187	39.37%	272	48.31%	
	Missing	13	2.74%	14	2.49%	
ASIA category at acute admission	A	192	40.42%	160	28.42%	<0.001
	B	73	15.37%	41	7.28%	
	C	79	16.63%	94	16.70%	
	D	92	19.37%	210	37.30%	
	Missing	39	8.21%	58	10.30%	
Lower extremity motor score	Median, IQR	0	(0–2)	17.5	(0–44)	<0.001
ISS score	Median, IQR	25	(17–30)	21	(16–29)	0.002
ASA class	1	24	5.05	20	3.55	0.020
	2	50	10.53	48	8.53	
	3	61	12.84	81	14.39	
	4	86	18.11	66	11.72	
	5	NR	NR	NR	NR	
	Unknown	NR	NR	NR	NR	
Neurological level of injury	Cervical	217	45.68%	278	49.38%	0.017
	Thoracic	141	29.68%	124	22.02%	
	Lumbar	54	11.37%	56	9.95%	
	Sacral	NR	NR	NR	NR	
	Missing	NR	NR	NR	NR	
Method of transport	Ground ambulance	258	54.32%	276	49.02%	0.221
	Air ambulance	48	10.11%	56	9.95%	
	Combination	NR	NR	NR	NR	
	Private transport	NR	NR	NR	NR	
	Other	46	9.68%	47	8.35%	
	Unknown or missing	113	23.79%	174	30.91%	

Abbreviations: ASA, American Society of Anesthesiologists; ASIA, American Spinal Injury Association; IQR, interquartile range; ISS, Injury Severity Score; NR, not reported for privacy reasons; SCI, spinal cord injury.

Surgical spinal cord decompression/stabilization occurred at a median of 25 (IQR 17–50) hours from injury. In the early decompression group, the median time to decompression/stabilization was 17 h (IQR 12–21). In the late decompression/stabilization group, the median time to decompression/stabilization was 48 (IQR 31–94) h. A small number of participants (7%, *n* = 75/1038) underwent very delayed decompression/stabilization at >7 days from SCI.

The median time from SCI to the 1‐year community follow‐up visit was 12 months (IQR 12–13); 63% (*n* = 650/1038) had abnormal bladder function. The sexual function question was completed by 515/1038 participants, and of those 69% (*n* = 355/515) reported abnormal sexual function. For motor function, the 1‐year FIM motor score was available for 575/1038 participants, and the median FIM motor score was 81 (IQR 57–88). The FIM mobility question was completed by 585 participants, and 48.2% (*n* = 282/585) of these participants were wheelchair dependent, and 6.5% (*n* = 38/585) occasionally required a wheelchair.

**FIGURE 2:**
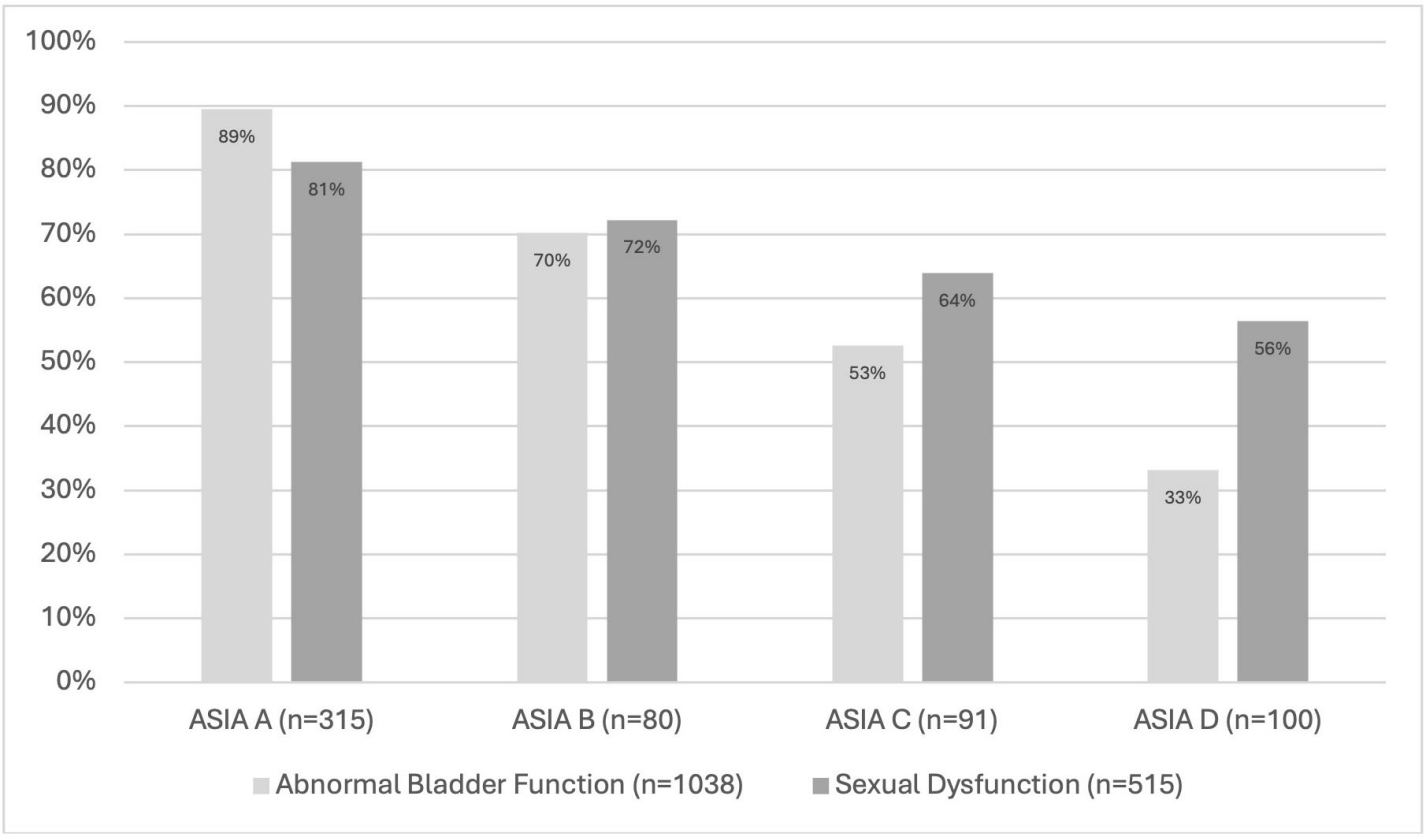
Absolute risk of bladder/sexual dysfunction 1‐year post‐SCI by ASIA Score.

In our multivariate logistic regression model (Table 2), time from SCI to surgical decompression was not significantly associated with bladder dysfunction 1‐year post‐SCI (OR 1.0, *p* = 0.38). Older age (OR 1.13, *p* = 0.01) and increasing ASIA category (ASIA A OR 16.35 *p* < 0.01, ASIA B OR 5.12 *p* < 0.01 and ASIA C 2.23 *p* < 0.01 versus ASIA D) were significantly associated with bladder dysfunction. The absolute risk of bladder or sexual dysfunction by ASIA score is shown in Figure 2. We did six sensitivity analyses to further assess this outcome, and in all cases the predictors were unchanged in direction of effect, and our primary outcome remained nonsignificant. We restricted the cohort to people with decompression within 7 days (*n* = 963) to remove the more extreme outliers in time to decompression (see Appendix S1). We restricted the cohort to those with ASIA B‐D (*n* = 686) to see if those with non‐complete injuries would have a greater potential for an improvement with more rapid decompression (Appendix S2). We redefined our primary outcome based only on people with a SCIM assessment at 1 year as the most specific assessment of bladder dysfunction (Appendix S3). We used lower extremity motor score (which has been previously studied in predicting NLUTD) instead of ASIA category in our multivariable model (Appendix S4). To look for a non‐linear effect of time to decompression, we did a logistic regression analysis where time from SCI to decompression was modelled as quintiles, rather than as a continuous outcome (Appendix S5). Finally, we repeated the primary analysis with a complete‐case approach and found the results were unchanged (Appendix S6).

**TABLE 2:** Multivariate logistic regression model for the primary outcome of abnormal bladder function.

Variable	OR (95% CI)	*p*‐value
Time from SCI to decompression (per 10‐h increase)	1.00 (1.00–1.01)	0.384
Age (per 10‐year increase)	1.13 (1.03–1.23)	0.011
Neurological level of injury		
Thoracic vs. cervical	1.32 (0.88–1.99)	0.183
Lumbar or sacral vs. cervical	0.89 (0.55–1.44)	0.639
Missing vs. cervical	1.23 (0.74–2.04)	0.434
Energy level of injury		
High vs. low/missing		
ASA classification		
2 vs. 1	1.18 (0.86–1.64)	0.309
3 vs. 1	0.81 (0.36–1.82)	0.614
4 or 5 vs. 1	1.00 (0.46–2.21)	0.991
Missing vs. 1	0.98 (0.45–2.13)	0.952
American Spinal Injury Association Classification (ASIA)		
ASIA (C vs. D)	2.23 (1.51–3.30)	<0.0001
ASIA (B vs. D)	5.12 (3.13–8.38)	<0.0001
ASIA (A vs. D)	16.35 (10.21–26.19)	<0.0001
ASIA (Missing vs. D)	3.33 (1.82–6.10)	<0.0001
Injury Severity Score (ISS)	1.01 (0.99–1.03)	0.289

Abbreviations: CI, confidence interval; OR, odds ratio; SCI, spinal cord injury.

For the secondary outcome of sexual dysfunction at 1 year (Table 3), our multivariable model found that time to surgical decompression was not significantly associated with sexual dysfunction (OR 1.02, *p* = 0.19). Sexual dysfunction was significantly associated with a thoracic versus cervical injury (OR 1.81, *p* = 0.04), and with an ASIA A versus ASIA D injury (OR 3.32, *p* < 0.01).

**TABLE 3:** Multivariate logistic regression for the secondary outcome of abnormal sexual function.

Variable	OR (95% CI)	*p*‐value
Time from SCI to decompression (per 10‐hour increase)	1.02 (0.99–1.04)	0.187
Age (per 10‐year increase)	1.10 (0.97–1.25)	0.130
Neurological level of Injury		
Thoracic vs Cervical	1.81 (1.02–3.23)	0.044
Lumbar or Sacral vs Cervical	1.59 (0.76–3.35)	0.219
Missing vs Cervical	0.70 (0.39–1.27)	0.247
Energy level of Injury		
High vs Low/Missing	1.22 (0.79–1.88)	0.377
ASA Classification		
2 vs 1	1.04 (0.46–2.37)	0.920
3 vs 1	1.00 (0.45–2.25)	0.997
4 or 5 vs 1	0.80 (0.36–1.75)	0.572
Missing vs 1	1.03 (0.45–2.36)	0.947
American Spinal Injury Association Classification (ASIA)		
ASIA (C vs. D)	1.36 (0.76–2.45)	0.3038
ASIA (B vs. D)	1.92 (0.94–3.89)	0.0718
ASIA (A vs. D)	3.32 (1.82–6.05)	<0.0001
ASIA (Missing vs. D)	3.46 (1.65–7.28)	0.001
Injury Severity Score (ISS)	1.00 (0.97–1.02)	0.682

Abbreviations: CI, confidence interval; OR, odds ratio; SCI, spinal cord injury.

The secondary outcome of motor function (using the FIM motor score at 1 year) was available for 575 participants (Table 4). On multivariable analysis there was again no significant association between time to surgical decompression and FIM motor score (beta −0.006, *p* = 0.22); however, age (Beta‐0.273, *p* < 0.0001), neurologic level of injury (Beta 6.596, *p* < 0.0001), ISS score (Beta‐0.201, *p* = 0.02), ASIA score (beta −8.15, *p* < 0.01), and energy level of injury (Beta 4.293, *p* = 0.309) were significant predictors.

**TABLE 4:** Multivariate linear regression for the secondary outcome of functional independence measure score.

Variable	Beta* (standard error)	*p*‐value	Interpretation
Time from SCI to decompression	−0.006 (0.005)	0.218	‐
Age	−0.273 (0.051)	<0.0001	Older age is associated with a lower FIM score
Neurological level of injury	6.596 (0.814)	<0.0001	Lower level lesions are associated with a higher FIM score
Energy level of injury	4.293 (1.868)	0.022	Lower energy injuries are associated with a higher FIM score
ASA Classification	−0.338 (1.633)	0.836	‐
ASIA category	−8.150 (0.667)	<0.0001	Higher ASIA categories are associated with a lower FIM score
ISS score	−0.201 (0.088)	0.023	Higher ISS score is associated with a lower FIM score

Abbreviations: ASA, American Society of Anesthesiologists; ASIA, American Spinal Injury Association; ISS, Injury Severity Score; SCI, spinal cord injury.

## 4 DISCUSSION

We did not find that time to operative decompression/stabilization was significantly associated with bladder, sexual or motor function 1 year after traumatic SCI. To our knowledge, this is the first large‐scale assessment of the association between intervention timing and eventual NLUTD function after SCI. One small study assessed the impact of ultra‐early (<8 h) decompression on bladder function after thoracic SCI and found that the SCIM score for bladder function improved in the 32 patients with ultra‐early decompression, compared to the 11 patients with late decompression.^17^ They found a significantly better SCIM score for Question 6 (shown in the eMethods) among people with ultra‐early decompression. However, it is hard to interpret what an improved median SCIM score means for this single question, which represents a combination of a patient's functional ability, continence, residual urine and choice of bladder management. Other variables in our model did have a statistically significant relationship with NLUTD such as injury completeness (measured by ASIA score at the time of the injury) and increasing age.

Age is an important determinant of lower urinary tract function, independent of an acute SCI, so it is not surprising it is also relevant for the recovery of people with a SCI.^18^ It is also correlated with reduced neuroplasticity, increased comorbidities and preexisting functional challenges and medical conditions which may limit rehabilitation. While our goal was not to create a prediction model, several are available for NLUTD that included variables relevant to our study. A study using 1250 people with SCI from the European Multicenter Study about Spinal Cord Injury found that the lower extremity motor score (LEMS) had a high area under the receiver‐operator curve (0.91) for predicting normal bladder function at 1‐year post‐SCI (defined as continence and postvoid residual urine <100 mL based on the SCIM questionnaire).^19^ Their cohort had a very similar proportion of patients with abnormal bladder function (68%, compared to 63% in our cohort), likely as a result of similar outcome questionnaires and outcome definitions. Other authors have similarly shown the importance of LEMS in predicting NLUTD. For example, data from a Chinese rehabilitation hospital demonstrated that LEMS, the H‐reflex of the soleus and time to rehabilitation were significantly associated with complete bladder emptying.^20^ The significance of ASIA category for NLUTD recovery in our study (Figure 2) is consistent with these studies as LEMS is highly correlated with ASIA category.^21^ A study using the National Spinal Cord Injury Model Systems database also found that ASIA category had a significant stepwise relationship with normal voiding 1 year after SCI.^22^

Similar to NLUTD, sexual dysfunction was not impacted by time to spinal stabilization/decompression. It was significantly more likely in those with thoracic versus cervical injuries (OR 1.81) and in more complete SCI (ASIA A vs. ASIA D, OR 3.46). As expected for a traumatic SCI study, our cohort was 76% male, and these results fit with our current understanding of the neuronal control of erections. If the patient has both light touch and pinprick sensation in the T11–L2 dermatomes they may still have psychogenic erections, and the presence of the bulbocavernosus reflex means they may still experience reflexogenic erections. Erectile function is generally improved in those with incomplete injuries and in those with complete lesions above T10.^23^ Intact sympathetic and parasympathetics in these people permit reflexogenic erections in as many as 70% of people with cervical and thoracic lesions at 6 months.^24^ Similar outcomes have been demonstrated in women, with more caudal injuries resulting in reduced likelihood of orgasm.^3^

Older age, a higher level of SCI, more associated injuries, a higher energy injury and more complete ASIA score at the time of the injury were significantly associated with worse motor function after 1 year. Most of the research on the impact of timing of SCI stabilization/decompression has focused on the recovery of motor function. Animal models have mixed results, with the majority suggesting there is improved blood flow, electrophysiological parameters and histologic changes when decompression is done as early as 6 h.^9^ However, clinical studies with limited levels of evidence initially reported variable results, with only some showing improved clinical and neurological outcomes, based on different definitions of ‘early’ surgical intervention (8, 24 or 72 h).^9^ However, more contemporary systematic reviews have identified a larger number of modern clinical studies, and a recent meta‐analysis calculated that early surgery (<24 h) was associated with a small but significant improvement in total motor score.^25^ Similarly, a large, pooled analysis of individual data from 1548 people with SCI drawn from four data sets found that early (<24 h) decompression/stabilization resulted in a significant improvement in motor function, and an almost 50% increased odds of improvement in ASIA category at 1 year.^26^ In keeping with the most recent literature, clinical care guidelines published in 2024 state that early surgery (<24 h) should be offered to adults with acute SCI (moderate evidence quality and strong recommendation).^27^ Limitations in comparing these studies to our work include the heterogeneity in outcomes, impact of missing data and selection bias, advances over time in the triage and management of SCI and the relatively modest change in the ASIA motor score (4–5 points/100), which is similar to the threshold of minimally important change for this score.^28^ None of these studies explored bladder or sexual function as an outcome.

Strengths of our study include the use of a large, multicentre, prospective registry, which is representative of a diverse traumatic SCI population. We conducted several additional analyses, which support our primary conclusion that time to surgical decompression/stabilization is not significantly related to NLUTD, with consistent odds ratios centred at approximately one. Limitations of our work must also be acknowledged. The topic of timing of surgical intervention after a traumatic SCI will always have significant selection bias because random and nonrandom factors almost certainly impact whether people undergo surgery early or late. We adjusted for several relevant variables; however, this does not remove the risk of residual confounding. The outcome definitions are consistent with those used in other studies assessing bladder function after SCI; however, they are not a precise assessment of bladder function. Ideally, validated patient reported outcome measures specific to the bladder, or clinical tests (such as urodynamic parameters) would be incorporated into the definition of abnormal bladder function, and more detailed questions around sexual function would be used. Some studies have suggested that better outcomes may result from ultra‐early spinal decompression (<8 h from SCI)^26^; our data set only had 26 patients who met this definition, so we were not able to provide a rigorous assessment of this group. Due to the small number (*n* = 7) of patients with sacral SCI, our results may not be generalizable to this specific group, who may have unique neurogenic bladder impairment due to the sacral innervation of the bladder. A more precise definition of NLUTD, or assessment of different specific aspects of NLUTD may identify more subtle differences in bladder function with early versus late intervention. Finally, there were data‐associated limitations with RHISCIR due to missing data.

## 5 CONCLUSION

We found that a shorter time to surgical stabilization/decompression after traumatic SCI was not associated with improved bladder, sexual or motor outcomes at 1 year. More complete injury (based on ASIA category) was associated with worse bladder and sexual function. These results provide reassurance to clinicians and people with SCI that appropriate timing of surgical intervention after SCI can be made based on clinical factors and existing guidelines, without evidence this will worsen long‐term bladder and sexual function.

## AUTHOR CONTRIBUTIONS

Conceptualization: BW. Methodology: BW, MP, JAM, CB. Data curation: JAM. Formal analysis: JAM. Writing—original draft: MP, BW. Writing—review and editing: JAM, CB. Supervision: BW. All authors reviewed and approved the final version of the manuscript.

## CONFLICT OF INTEREST STATEMENT

The authors declare no conflicts of interest.

## PATIENT CONSENT STATEMENT

Patients included in this study provided written informed consent.

## Supporting information


eMethods
**Appendix 1.** Logistic regression predicting abnormal bladder function, cohort restricted to participants with decompression within 7 days of injury (n = 963 in this model).
**Appendix 2.** Logistic regression predicting abnormal bladder function, excluding participants with ASIA = A (n = 686 in this model).
**Appendix 3.** Logistic regression predicting SCIM defined abnormal bladder function (n = 435).
**Appendix 4.** Logistic regression analysis predicted abnormal bladder function with lower extremity motor score, instead of ASIA category.
**Appendix 5.** Logistic regression analysis predicted abnormal bladder function, with time from SCI to decompression modelled as quintiles to determine if there are non‐linear effects.
**Appendix 6.** Logistic regression analysis predicted abnormal bladder function, including only patients with complete‐case data (174 patients with missing data were excluded).

## Data Availability

The data set from this study is held and managed by Praxis and available through an online request process.
