# Supplementary material for: The impact of the timing of spinal decompression on urinary and sexual function after acute spinal cord injury
Source: BJUI Compass. 2026 Jan 30;7(2):e70163. doi: 10.1002/bco2.70163 (PMC12858374; doi:10.1002/bco2.70163)
Supplement: Supplementary file 1 — eMethods Appendix 1. Logistic regression predicting abnormal bladder function, cohort restricted to participants with decompression within 7 days of injury (n = 963 in this model). Appendix 2. Logistic regression predicting abnormal bladder function, excluding participants with ASIA = A (n = 686 in this model). Appendix 3. Logistic regression predicting SCIM defined abnormal bladder function (n = 435). Appendix 4. Logistic regression analysis predicted abnormal bladder function with lower extremity motor score, instead of ASIA category. Appendix 5. Logistic regression analysis predicted abnormal bladder function, with time from SCI to decompression modelled as quintiles to determine if there are non‐linear effects. Appendix 6. Logistic regression analysis predicted abnormal bladder function, including only patients with complete‐case data (174 patients with missing data were excluded). [file BCO2-7-e70163-s001.docx]

**Online Appendix**

**eMethods**

The primary outcome of abnormal bladder function was determined using these questions from the 1 year community follow-up questionnaire. If any of the questions were answered with a response categorized as abnormal, they were considered to have abnormal bladder function.

| **FIM Question** |  |
| --- | --- |
| Sphincter Control: g) Bladder Management  Answer options  *No Helper*  7 Complete Independence (Timely, Safely)  6 Modified Independence (Device)  *Helper - Complete Dependence*  5 Supervision  4 Minimal Assistance (Subject = 75% + )  3 Moderate Assistance (Subject = 50% + )  *Helper - Complete Dependence*  2 Maximal Assistance (Subject = 25% + )  1 Total Assistance (Subject = 0% + ) | Answer of 1-6 considered abnormal bladder function. |
| **SCIM question** |  |
| 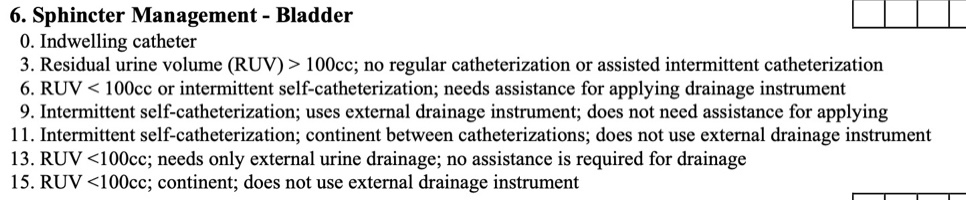 | Answer 0-13 = abnormal bladder function |
| **Bladder management questions** |  |
| *Please think about the way you empty your bladder.*  (a) Use of an indwelling catheter  i) Yes → Please go to question 7a  ii) No → Please also answer questions 6b and 6c | Answer: (a) i) = Abnormal bladder function |
| (b) Intermittent catheterization  i) I need total assistance  ii) I do it myself with assistance (self-catheterization)  iii) I do it myself without assistance (self-catheterization)  iv) I do not use it | Answer (b) i), ii), or iii) = Abnormal bladder function |
| (c) Use of external drainage instruments (e.g., condom catheter,  diapers, sanitary napkins)  i) I need total assistance for using them  ii) I need partial assistance for using them  iii) I use them without assistance  iv) I am continent with urine and do not use external drainage instruments | Answer (c) i), ii), or iii) = Abnormal bladder function |
| **Urinary incontinence question** |  |
| 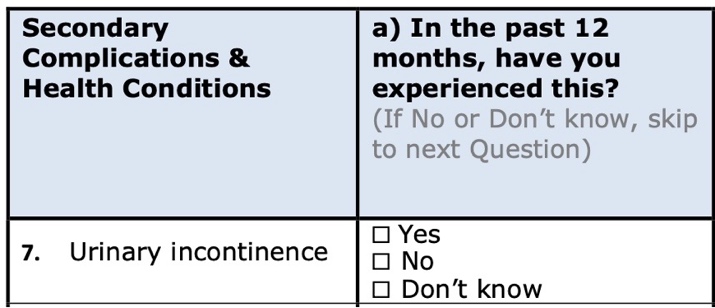  * Urinary incontinence (Urine leakage, catheter bypassing.) | Answer “Yes” = Abnormal bladder function |

Secondary outcomes

*Sexual dysfunction*

| 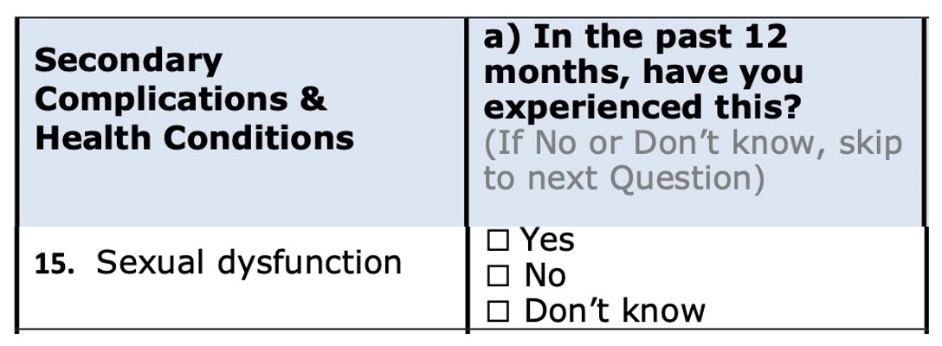  **Sexual dysfunction** (This includes dissatisfaction with sexual functioning. Causes for dissatisfaction can be decreased sensation, changes in body image, difficulty in movement, and problems with bowel or bladder, like infections.) | Answer “Yes” = Abnormal sexual function |
| --- | --- |

*FIM Motor Score Questions*

| - Self-Care:   - Eating   - Grooming   - Bathing   - Dressing (upper body)   - Dressing (lower body)   - Toileting - Sphincter Control:   - Bladder management   - Bowel management - Transfers:   - Transfers (bed/chair/wheelchair)   - Transfers (toilet)   - Transfers (tub/shower) - Locomotion:   - Walking/Wheelchair   - Stair Climbing | Scoring for each question:  No Helper  7 Complete Independence (Timely, Safely)  6 Modified Independence (Device)  Helper - Complete Dependence  5 Supervision  4 Minimal Assistance (Subject = 75% + )  3 Moderate Assistance (Subject = 50% + )  Helper - Complete Dependence  2 Maximal Assistance (Subject = 25% + )  1 Total Assistance (Subject = 0% + ) |
| --- | --- |

*Sensitivity Analyses*

First, we excluded participants with decompression >7 days after injury. Second, we excluded participants with complete injury at presentation (ASIA A). Third, for the subgroup of participants with an SCIM assessment at one-year, we used SCIM alone to define abnormal bladder function. Fourth, we used lower extremity motor function instead of ASIA category (both could not be included as they were collinear, r=-0.82, p<0.0001) in the multivariable model, to ensure that our primary outcome was unchanged. Fifth, for each study outcome, we conducted a sensitivity analysis where time from injury to decompression was modeled as a categorical variable to look for non-linear changes, with time categorized by quintile (Quintile 1 = 0-14, 2 = 15-21, 3 = 22-30, 4 = 31-65, and 5 = ≥66 hours). Finally, we conducted a complete case analysis because it yields potentially better, unbiased estimates when data are missing completely at random.

*Statistical methods*

Missing data was handled as follows: we combined missing energy level (n=27) with the low energy group to create a single reference group. Median imputation was used to impute ISS score where missing. To evaluate the impact of this missing data for ISS, the models were repeated without ISS to confirm there was no change in the exposure variable’s significance or direction of effect (results not shown). For the other variables, missing data was explored and found to be missing at random, and was modelled as its own category.

For our multivariable models, we evaluated multicollinearity using variance inflation factors; all VIFs were <3. Model fit was evaluated using the Hosmer-Lemeshow test (logistic regression), and using residual plots (linear regression).

**Appendix 1. Logistic regression predicting abnormal bladder function, cohort restricted to participants with decompression within 7 days of injury (n = 963 in this model)**

| **Variable** | **All covariates** | | | |
| --- | --- | --- | --- | --- |
|  | **OR** | **Lower 95% CI** | **Upper 95% CI** | **p-values** |
| Time from SCI to decompression (per 10-hour increase) | 0.96 | 0.92 | 1.01 | 0.143 |
| Age (per 10-year increase) | 1.15 | 1.04 | 1.26 | 0.005 |
| Neurological level of injury (T vs C) | 1.27 | 0.84 | 1.93 | 0.259 |
| Neurological level of injury (L or S vs C) | 0.89 | 0.54 | 1.46 | 0.639 |
| Neurological level of injury (missing vs C) | 1.39 | 0.80 | 2.40 | 0.238 |
| Energy level of injury (High vs low or missing) | 1.11 | 0.79 | 1.56 | 0.552 |
| ASA (2 vs 1) | 0.82 | 0.36 | 1.88 | 0.641 |
| ASA (3 vs 1) | 0.83 | 0.37 | 1.87 | 0.649 |
| ASA (4 or 5 vs 1) | 0.87 | 0.39 | 1.93 | 0.726 |
| ASA (Missing vs 1) | 0.94 | 0.46 | 1.95 | 0.875 |
| ASIA (C vs D) | 2.25 | 1.49 | 3.41 | 0.0001 |
| ASIA (B vs D) | 5.23 | 3.12 | 8.77 | <.0001 |
| ASIA (A vs D) | 16.69 | 10.21 | 27.28 | <.0001 |
| ASIA (Missing vs D) | 3.26 | 1.70 | 6.23 | 0.0004 |
| ISS score | 1.01 | 0.99 | 1.02 | 0.560 |

**Appendix 2. Logistic regression predicting abnormal bladder function, excluding participants with ASIA = A (n = 686 in this model)**

| **Variable** | **All covariates** | | | |
| --- | --- | --- | --- | --- |
|  | **OR** | **Lower 95% CI** | **Upper 95% CI** | **p-values** |
| Time from SCI to decompression (per 10-hour increase) | 1.00 | 0.99 | 1.01 | 0.460 |
| Age (per 10-year increase) | 1.10 | 1.00 | 1.21 | 0.055 |
| Neurological level of injury (T vs C) | 1.17 | 0.71 | 1.90 | 0.541 |
| Neurological level of injury (L or S vs C) | 0.88 | 0.53 | 1.48 | 0.636 |
| Neurological level of injury (missing vs C) | 1.25 | 0.73 | 2.13 | 0.419 |
| Energy level of injury (High vs low or missing) | 1.21 | 0.85 | 1.73 | 0.285 |
| ASA (2 vs 1) | 0.83 | 0.35 | 1.96 | 0.671 |
| ASA (3 vs 1) | 1.09 | 0.47 | 2.53 | 0.833 |
| ASA (4 or 5 vs 1) | 1.18 | 0.51 | 2.73 | 0.691 |
| ASA (Missing vs 1) | 0.85 | 0.40 | 1.79 | 0.662 |
| ASIA (C vs D) | 2.29 | 1.54 | 3.39 | <.0001 |
| ASIA (B vs D) | 5.28 | 3.22 | 8.67 | <.0001 |
| ASIA (Missing vs D) | 3.21 | 1.74 | 5.95 | 0.0002 |
| ISS score | 1.00 | 0.98 | 1.02 | 0.906 |

**Appendix 3. Logistic regression predicting SCIM defined abnormal bladder function (n = 435)**

| **Variable** | **All covariates** | | | |
| --- | --- | --- | --- | --- |
|  | **OR** | **Lower 95% CI** | **Upper 95% CI** | **p-values** |
| Time from SCI to decompression (per 10-hour increase) | 1.00 | 0.97 | 1.02 | 0.707 |
| Age (per 10-year increase) | 1.04 | 0.89 | 1.20 | 0.634 |
| Neurological level of injury (T vs C) | 2.18 | 1.06 | 4.46 | 0.034 |
| Neurological level of injury (L or S vs C) | 1.31 | 0.58 | 2.96 | 0.523 |
| Neurological level of injury (missing vs C) | 1.47 | 0.70 | 3.09 | 0.305 |
| Energy level of injury (High vs low or missing) | 0.94 | 0.55 | 1.59 | 0.812 |
| ASA (2 vs 1) | 0.49 | 0.19 | 1.25 | 0.137 |
| ASA (3 vs 1) | 0.47 | 0.19 | 1.17 | 0.104 |
| ASA (4 or 5 vs 1) | 0.58 | 0.24 | 1.43 | 0.237 |
| ASA (Missing vs 1) | 0.43 | 0.17 | 1.08 | 0.072 |
| ASIA (C vs D) | 2.84 | 1.45 | 5.58 | 0.0024 |
| ASIA (B vs D) | 6.56 | 2.99 | 14.38 | <.0001 |
| ASIA (A vs D) | 36.08 | 15.15 | 85.94 | <.0001 |
| ASIA (Missing vs D) | 2.89 | 1.29 | 6.50 | 0.0101 |
| ISS score | 1.01 | 0.98 | 1.04 | 0.601 |

**Appendix 4. Logistic regression analysis predicted abnormal bladder function with lower extremity motor score, instead of ASIA category.**

| **Variable** | **All covariates** | | | |
| --- | --- | --- | --- | --- |
|  | **OR** | **Lower 95% CI** | **Upper 95% CI** | **p-values** |
| Time from SCI to decompression (per 10-hour increase) | 1.01 | 1.00 | 1.02 | 0.265 |
| Age (per 10-year increase) | 1.08 | 0.99 | 1.18 | 0.072 |
| Neurological level of injury (T vs C) | 1.52 | 1.04 | 2.22 | 0.032 |
| Neurological level of injury (L or S vs C) | 1.03 | 0.65 | 1.62 | 0.914 |
| Neurological level of injury (missing vs C) | 1.05 | 0.70 | 1.58 | 0.804 |
| Energy level of injury (High vs low or missing) | 1.22 | 0.90 | 1.67 | 0.207 |
| ASA (2 vs 1) | 0.93 | 0.42 | 2.05 | 0.860 |
| ASA (3 vs 1) | 1.35 | 0.63 | 2.91 | 0.445 |
| ASA (4 or 5 vs 1) | 1.11 | 0.52 | 2.36 | 0.796 |
| ASA (Missing vs 1) | 1.13 | 0.57 | 2.26 | 0.726 |
| Lower extremity motor score | 0.96 | 0.95 | 0.97 | <.0001 |
| ISS score | 1.03 | 1.01 | 1.04 | 0.002 |

**Appendix 5. Logistic regression analysis predicted abnormal bladder function, with time from SCI to decompression modelled as quintiles to determine if there are non-linear effects.**

| **Variable** | **All covariates** | | | |  |
| --- | --- | --- | --- | --- | --- |
|  | **OR** | **Lower 95% CI** | **Upper 95% CI** | **p-values** | |
| Time from injury to decompression (Quintile 2 vs Quintile 1) | 0.89 | 0.55 | 1.44 | 0.637 | |
| Time from injury to decompression (Quintile 3 vs Quintile 1) | 1.03 | 0.64 | 1.65 | 0.899 | |
| Time from injury to decompression (Quintile 4 vs Quintile 1) | 1.05 | 0.65 | 1.69 | 0.839 | |
| Time from injury to decompression (Quintile 5 vs Quintile 1) | 0.83 | 0.51 | 1.33 | 0.431 | |
| Age (per 10-year increase) | 1.14 | 1.04 | 1.25 | 0.007 | |
| Neurological level of injury (T vs C) | 1.31 | 0.87 | 1.98 | 0.197 | |
| Neurological level of injury (L or S vs C) | 0.90 | 0.56 | 1.45 | 0.654 | |
| Neurological level of injury (missing vs C) | 1.25 | 0.75 | 2.09 | 0.385 | |
| Energy level of injury (High vs low or missing) | 1.18 | 0.86 | 1.64 | 0.305 | |
| ASA (2 vs 1) | 0.80 | 0.36 | 1.81 | 0.598 | |
| ASA (3 vs 1) | 1.02 | 0.46 | 2.25 | 0.959 | |
| ASA (4 or 5 vs 1) | 0.96 | 0.44 | 2.10 | 0.926 | |
| ASA (Missing vs 1) | 0.92 | 0.45 | 1.86 | 0.809 | |
| ASIA (C vs D) | 2.15 | 1.45 | 3.19 | 0.0001 | |
| ASIA (B vs D) | 5.02 | 3.04 | 8.28 | <.0001 | |
| ASIA (A vs D) | 15.87 | 9.86 | 25.53 | <.0001 | |
| ASIA (Missing vs D) | 3.40 | 1.85 | 6.24 | <.0001 | |
| ISS score | 1.01 | 0.99 | 1.03 | 0.325 | |

**Appendix 6. Logistic regression analysis predicted abnormal bladder function, including only patients with complete case data (174 patients with missing data were excluded).**

|  | **OR** | **Lower 95% CI** | **Upper 95% CI** | **p-value** |
| --- | --- | --- | --- | --- |
| Time from SCI to decompression (per 10-hour increase) | 1.01 | 0.99 | 1.02 | 0.33 |
| Age (per 10-year increase) | 1.12 | 1.01 | 1.24 | 0.03 |
| Neurological level of injury (T vs C) | 1.30 | 0.85 | 1.98 | 0.23 |
| Neurological level of injury (L or S vs C) | 0.87 | 0.53 | 1.43 | 0.60 |
| Energy level of injury (High vs low or missing) | 1.18 | 0.82 | 1.71 | 0.37 |
| ASA (2 vs 1) | 0.43 | 0.16 | 1.19 | 0.10 |
| ASA (3 vs 1) | 0.76 | 0.29 | 1.97 | 0.57 |
| ASA (4 or 5 vs 1) | 0.73 | 0.28 | 1.94 | 0.53 |
| ASA (Missing vs 1) | 0.63 | 0.26 | 1.48 | 0.29 |
| ASIA (C vs D) | 2.46 | 1.62 | 3.74 | <.0001 |
| ASIA (B vs D) | 6.09 | 3.64 | 10.19 | <.0001 |
| ASIA (A vs D) | 19.5 | 11.77 | 32.37 | <.0001 |
| ISS score | 1.01 | 0.99 | 1.03 | 0.58 |
